# Development and validation of prediction models for the prognosis of colon cancer with lung metastases: a population-based cohort study

**DOI:** 10.3389/fendo.2023.1073360

**Published:** 2023-07-31

**Authors:** Zhenyu Ma, Shuping Yang, Yalin Yang, Jingran Luo, Yixiao Zhou, Huiyong Yang

**Affiliations:** School of Medicine, Huaqiao University, Quanzhou, China

**Keywords:** colon cancer, lung metastases, prognosis, prediction model, nomogram, decision curve analysis, SEER

## Abstract

**Background:**

Current studies on the establishment of prognostic models for colon cancer with lung metastasis (CCLM) were lacking. This study aimed to construct and validate prediction models of overall survival (OS) and cancer-specific survival (CSS) probability in CCLM patients.

**Method:**

Data on 1,284 patients with CCLM were collected from the Surveillance, Epidemiology, and End Results (SEER) database. Patients were randomly assigned with 7:3 (stratified by survival time) to a development set and a validation set on the basis of computer-calculated random numbers. After screening the predictors by the least absolute shrinkage and selection operator (LASSO) and multivariate Cox regression, the suitable predictors were entered into Cox proportional hazard models to build prediction models. Calibration curves, concordance index (C-index), time-dependent receiver operating characteristic (ROC) curves, and decision curve analysis (DCA) were used to perform the validation of models. Based on model-predicted risk scores, patients were divided into low-risk and high-risk groups. The Kaplan–Meier (K-M) plots and log-rank test were applied to perform survival analysis between the two groups.

**Results:**

Building upon the LASSO and multivariate Cox regression, six variables were significantly associated with OS and CSS (i.e., tumor grade, AJCC T stage, AJCC N stage, chemotherapy, CEA, liver metastasis). In development, validation, and expanded testing sets, AUCs and C-indexes of the OS and CSS prediction models were all greater than or near 0.7, which indicated excellent predictability of models. On the whole, the calibration curves coincided with the diagonal in two models. DCA indicated that the models had higher clinical benefit than any single risk factor. Survival analysis results showed that the prognosis was worse in the high-risk group than in the low-risk group, which suggested that the models had significant discrimination for patients with different prognoses.

**Conclusion:**

After verification, our prediction models of CCLM are reliable and can predict the OS and CSS of CCLM patients in the next 1, 3, and 5 years, providing valuable guidance for clinical prognosis estimation and individualized administration of patients with CCLM.

## Introduction

Colorectal cancer (CRC) has been reported as a threat to human health worldwide and a burden to society and families (1). In the past 30 years, the global prevalence of CRC was in the rising trend year after year and the morbidity and mortality have doubled or more than doubled in a dozen or so world regions (2). According to the statistics of global cancer released by GLOBALCAN in 2020, the incidence of CRC in both sexes was in the third rank and the mortality was in the second rank (3). In countries with a middle and high human development index (HDI), the increase of morbidity and mortality especially was in the young population, which was related to smoking, alcohol consumption, low calcium and fiber diets, obesity, and physical inactivity (4).

At present, CRC was seen as a whole cohort to analyze the prognostic factors in many studies (5–7). However, colon and rectal cancers differ in incidence, mortality, and patterns of distant metastasis. Evidence has shown that the incidence of colon cancer (CC) is higher than that of rectal cancer. The clinical prognosis and distant metastasis preference in CC patients were also different with rectal cancer (8, 9). Therefore, it was necessary to deem CC patients as a unique subset to further study. Among all distant metastases of CC, the presence rate of lung metastatic sites accounted for around 30%, second only to liver metastases (8). Furthermore, a systematic pan-cancer analysis revealed that colon cancer ranked first in the distribution of primary cancer in cases with pulmonary metastasis (10). Universally known, distant organ metastatic spread could contribute to poorer prognosis for CC patients (11). Unless it could be surgically removed, the prognosis for colon cancer with lung metastases (CCLM) was poor (12). At present, the American Joint Committee on Cancer (AJCC) tumor node metastasis (TNM) stage system was broadly applied for prognosis prediction of CCLM patients (13). Nevertheless, with the effects on prognosis of other clinical risk factors (e.g., age, tumor grade, and chemotherapy), the AJCC TNM stage system could not provide personalized prognostic reference of CCLM patients well. Therefore, it was necessary to evaluate the prognostic factors and construct prediction models for patients with CCLM.

In this study, we collected a large amount of clinical data of CCLM from the Surveillance, Epidemiology, and End Results (SEER) database. By the retrospective analysis for these data, we constructed and validated the 1-, 3-, 5-year overall survival and cancer-specific survival prediction models. It will provide new ideas and help for the clinical personalized prognostic evaluation of CCLM.

## Materials and methods

### Patients

**Figure 1:**
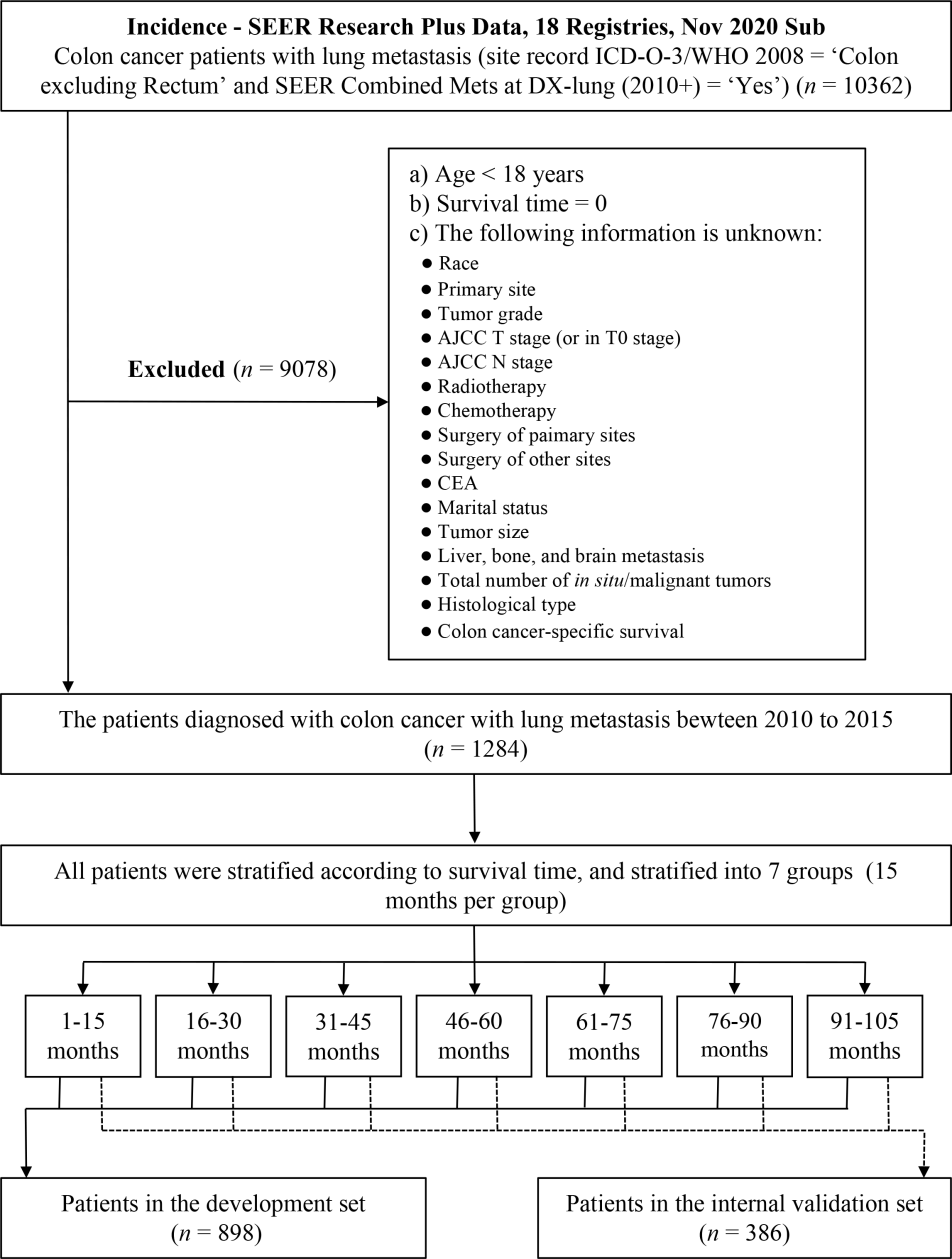
Flowchart of the patient screening.

The Surveillance, Epidemiology, and End Results (SEER) database was an open-access cancer database covering around 30% of the United States (US) population, which recorded information about cancer incidence, treatment, and survival (14). The data of patients diagnosed with colon cancer with lung metastasis (CCLM) were collected from the SEER database using SEER*Stat software (version 8.4.0; https://seer.cancer.gov/seerstat/ ). Patients with CCLM were identified using site record ICD-O-3/WHO 2008 = ‘Colon excluding Rectum’ and SEER Combined Mets at DX-lung (2010+) = ‘Yes’. The data of the CC-related variables were downloaded from the database “Incidence - SEER Research Plus Data, 18 Registries, Nov 2020 Sub (2000–2018)” (i.e., age, sex, race, primary site, tumor grade, AJCC T stage, AJCC N stage, radiotherapy, chemotherapy, carcinoembryonic antigen (CEA), marital status, tumor size, bone metastasis, brain metastasis, liver metastasis, total number of *in situ*/malignant tumors for patient, surgery of primary sites, surgery of distant lymph nodes or other tissues or organs beyond the primary sites, histologic type, survival months, overall survival status, and cancer-specific survival status). Patients with age <18 years, survival months = 0, or unclear or missing relevant clinical information were excluded from the analysis. All patients were randomly assigned 7:3 to a development set and a validation set on the basis of computer-calculated random numbers. Randomization was stratified by survival time (15). The flowchart of the patients screening is shown in **Figure 1**.

### Development and validation of prediction models

The study was a population-based retrospective cohort study. Number and percentage (*N*, %) were used to describe the categorical data, and chi-square test was used to compare the difference between development and validation sets. According to the previous relevant studies and clinical experience (7, 12, 16), 18 independent variables were considered as candidate predictors, including age, sex, race, primary site, tumor grade, AJCC T stage, AJCC N stage, radiotherapy, chemotherapy, CEA, marital status, tumor size, bone metastasis, brain metastasis, liver metastasis, tumor number, surgery sites, and histologic type. Among them, the predictor “surgery sites” was defined according to the surgery of primary sites and distant lymph nodes or other tissues or organs beyond the primary sites. The least absolute shrinkage and selection operator (LASSO) regression analysis was employed to screen out suitable prognostic predictors from these 18 clinical variables (17). Multivariable Cox regression analysis was used to determine whether the selected variables were significantly colon-cancer-associated, and then the predictors with *P* < 0.05 were entered into Cox proportional hazard models to construct prediction models in CCLM patients, presented as the nomograms (18). The prediction outcomes were 1-, 3-, and 5-year overall survival (OS) and cancer-specific survival (CSS) probability.

The time-dependent receiver operating characteristic (ROC) curves and concordance index (C-index) were used to evaluate the predictability of the models. The areas under ROC curves (AUCs) and C-index ranged from 0.5 to 1, and the values over 0.7 indicated nice predictability. Calibration curve plots were used to assess the difference degree between the predicted and actual risks. Decision curve analysis (DCA) was performed to evaluate the clinical benefit and utility of the constructed prediction models. Furthermore, we divided all the patients into low-risk and high-risk levels according to the model-predicted risk score. The patients with a risk score higher than the median were assigned into the high-risk group, and the rest were assigned into the low-risk group. The Kaplan–Meier (K-M) plots and log-rank test were used to perform survival analysis to compare the survival difference between the two groups. The hazard ratio (HR) with 95% confidence interval (CI) was calculated. It was regarded as a significant difference when *P*-value < 0.05. All the statistical analysis was in R software (version 4.2.1; https://www.r-project.org/) with the “rms,” “glmnet,” “survival,” “survminer,” “timeROC,” and “ggDCA” packages.

## Results

### Baseline characteristics

The clinical characteristics of all the patients are shown in **Table 1**. A total of 1,284 patients between 2010 and 2015 with CCLM were included in our study, 898 of which were in the development set and the others were in the validation set. The chi-square test showed no significant difference between all the variables in the development and validation sets except primary site and liver metastasis (*P* < 0.05). Among all patients, 50.5% were older than 65 years, 51.1% were men, and 71.8% were white people. For the tumor primary sites, 17.4% patients were in the ascending colon, 9.1% in the transverse colon, 6.6% in the descending colon, 32.3% in the sigmoid colon, and 34.5% in other sites (i.e., cecum, appendix, hepatic flexure of colon, splenic flexure of colon, and overlapping lesion of colon). In all 1,284 individuals, 915 (71.3%) underwent chemotherapy and 75 (5.8%) underwent radiotherapy. Furthermore, the median time of the entire cohort was 17 months (interquartile range [IQR], 6–32 months), the OS rate through the ending of follow-up was 11.7%, and the CSS rate was 17.4%.

**Table 1:** Baseline characteristics of all 1,284 patients from SEER.

Variable	Overall patients (*N* = 1,284)	Development set (*N* = 898)	Validation set (*N* = 386)	χ2	*P* value
**Age**				0.29	0.59
<65	635 (49.5%)	449 (50.0%)	186 (48.2%)		
≥65	649 (50.5%)	449 (50.0%)	200 (51.8%)		
**Sex**				1.40	0.24
Female	628 (48.9%)	429 (47.8%)	199 (51.6%)		
Male	656 (51.1%)	469 (52.2%)	187 (48.4%)		
**Race**				2.69	0.26
White	922 (71.8%)	635 (70.7%)	287 (74.4%)		
Black	219 (17.1%)	155 (17.3%)	64 (16.6%)		
Others^*^	143 (11.1%)	108 (12.0%)	35 (9.1%)		
**Primary site**				10.26	0.04
Ascending colon	224 (17.4%)	149 (16.6%)	75 (19.4%)		
Transverse colon	117 (9.1%)	73 (8.1%)	44 (11.4%)		
Descending colon	85 (6.6%)	57 (6.3%)	28 (7.3%)		
Sigmoid colon	415 (32.3%)	312 (34.7%)	103 (26.7%)		
Others^**^	443 (34.5%)	307 (34.2%)	136 (35.2%)		
**Tumor grade**				0.63	0.43
I–II	939 (73.1%)	663 (73.8%)	276 (71.5%)		
III–IV	345 (26.9%)	235 (26.2%)	110 (28.5%)		
**AJCC T stage**				1.65	0.40
T1–T3	725 (56.5%)	518 (57.7%)	207 (53.6%)		
T4	559 (43.5%)	380 (42.3%)	179 (46.4%)		
**AJCC N stage**				< 0.01	1
N0–N1	672 (52.3%)	470 (52.3%)	202 (52.3%)		
N2	612 (47.7%)	428 (47.7%)	184 (47.7%)		
**Radiotherapy**				0.28	0.60
No/unknown	1,209 (94.2%)	843 (93.9%)	366 (94.8%)		
Yes	75 (5.8%)	55 (6.1%)	20 (5.2%)		
**Chemotherapy**				0.35	0.55
No/unknown	369 (28.7%)	263 (29.3%)	106 (27.5%)		
Yes	915 (71.3%)	635 (70.7%)	280 (72.5%)		
**CEA**				1.31	0.25
Negative	232 (18.1%)	170 (18.9%)	62 (16.1%)		
Positive	1,052 (81.9%)	728 (81.1%)	324 (83.9%)		
**Marital status**				0.37	0.54
Married	697 (54.3%)	482 (53.7%)	215 (55.7%)		
Unmarried	587 (45.7%)	416 (46.3%)	171 (44.3%)		
**Tumor size**				2.00	0.37
<5 cm	520 (40.5%)	374 (41.6%)	146 (37.8%)		
5–10 cm	117 (9.1%)	83 (9.2%)	34 (8.8%)		
>10 cm	647 (50.4%)	441 (49.1%)	206 (53.4%)		
**Bone metastasis**				0.03	0.87
No	1,197 (93.2%)	836 (93.1%)	361 (93.5%)		
Yes	87 (6.8%)	62 (6.9%)	25 (6.5%)		
**Brain metastasis**				1.48	0.22
No	1,256 (97.8%)	875 (97.4%)	381 (98.7%)		
Yes	28 (2.2%)	23 (2.6%)	5 (1.3%)		
**Liver metastasis**				7.52	0.01
No	449 (35.0%)	336 (37.4%)	113 (29.3%)		
Yes	835 (65.0%)	562 (62.6%)	273 (70.7%)		
**Tumor number**				2.02	0.16
Single	1,025 (79.8%)	707 (78.7%)	318 (82.4%)		
Multiple	259 (20.2%)	191 (21.3%)	68 (17.6%)		
**Surgery sites**				0.32	0.85
Only primary site	1,026 (79.9%)	714 (79.5%)	312 (80.8%)		
Primary and other sites^†^	255 (19.9%)	182 (20.3%)	73 (18.9%)		
No surgery	3 (0.2%)	2 (0.2%)	1 (0.3%)		
**Histological type**				2.10	0.15
Adenocarcinoma	1,170 (91.1%)	811 (90.3%)	359 (93.0%)		
Others^‡^	114 (8.9%)	87 (9.7%)	27 (7.0%)		

^*^Asian or Pacific Islander, American Indian/Alaska Native. ^**^Cecum, appendix, hepatic flexure of colon, splenic flexure of colon, and overlapping lesion of colon. ^†^Surgery of other sites describes the surgical removal of distant lymph nodes or other tissues or organs beyond the primary site. ^‡^Cystic, mucinous, and serous neoplasms, ductal and lobular neoplasm, complex epithelial neoplasms. CEA, carcinoembryonic antigen.

Based on the presence or absence of extrapulmonary metastases to the bone, brain, and liver, we divided all patients into two cohorts, CCLM without extrapulmonary metastases (n = 418) and CCLM with extrapulmonary metastases (n = 866). We compared the differences in clinicopathological characteristics and treatment options between the two cohorts, as shown in **Supplementary Table S1**. Patients with extrapulmonary metastatic sites tended to have an N2 stage and a positive CEA serum level. In addition, more patients with extrapulmonary metastatic sites underwent radiotherapy and resection of non-primary sites.

### Development of prediction models

**Figure 2:**
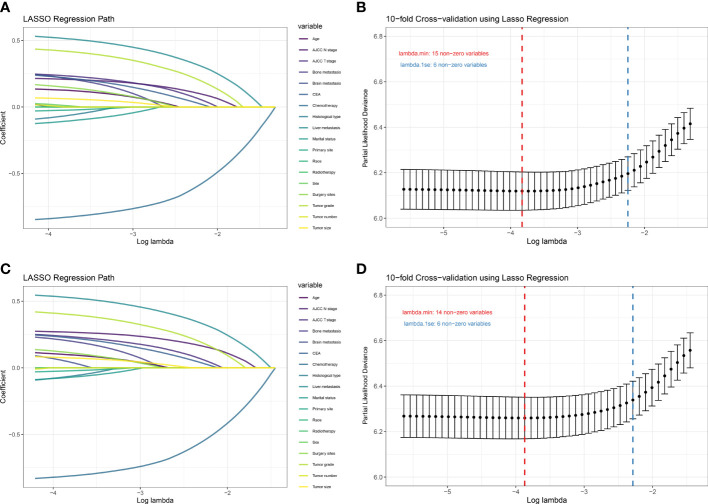
Plot of LASSO coefficient profiles of the 18 candidate predictors for OS **(A)** and CSS **(C)**. Plot of partial likelihood deviance for OS **(B)** and CSS **(D)**; the left vertical dotted lines were drawn at the values of log (λ) chosen by minimum criteria, and the right vertical dotted lines were drawn at the values of log (λ) chosen by one standard error of the minimum criteria.

**Figure 3:**
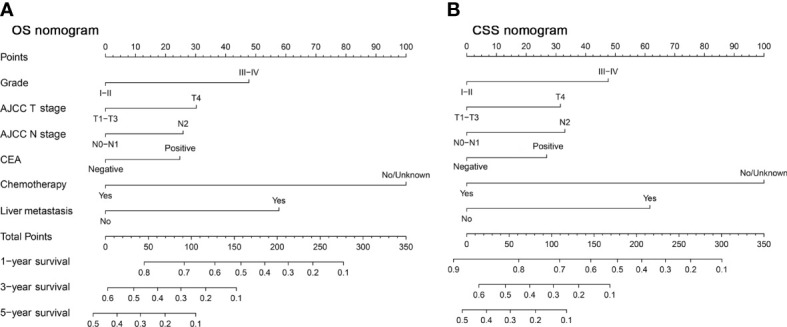
Nomogram for predicting the OS **(A)** and CSS **(B)** of colon cancer with lung metastases.

There were 18 independent candidate variables (**Table 1**) included in the LASSO regression model (**Figures 2A, C**). When the partial-likelihood deviance was the lowest, 15 and 14 variables were prognostic factors for OS and CSS, respectively. For getting simpler, more interpretable models, we used the log (λ) values chosen by one standard error of the minimum criteria and selected the variables with non-zero coefficients. Finally, we selected six predictors (i.e., tumor grade, AJCC T stage, AJCC N stage, chemotherapy, CEA, liver metastasis) in the multivariable Cox regression analysis for OS and CSS (**Figures 2B, D**). The predictor was identified as a risk factor for death when the corresponding coefficient was >0 or when the HR value was significantly >1 or, conversely, as a protective factor. Based on the multivariable Cox regression for OS, tumor grade (β = 0.48; HR = 1.62), AJCC T stage, (β = 0.30; HR = 1.35), AJCC N stage (β = 0.26; HR = 1.30), chemotherapy (β = -1.01; HR = 0.37), CEA (β = 0.25; HR = 1.28), and liver metastasis (β = 0.58; HR = 1.79) were deemed to be significantly associated with the OS in the development set. Based on the multivariable Cox regression for CSS, tumor grade (β = 0.46; HR = 1.59), AJCC T stage, (β = 0.31; HR = 1.36), AJCC N stage (β = 0.32; HR = 1.38), chemotherapy (β = -0.98; HR = 0.38), CEA (β = 0.26; HR = 1.30), and liver metastasis (β = 0.60; HR = 1.82) were deemed to be significantly associated with the CSS in the development set (**Table 2**). These significant variables above were incorporated into the final 1-, 3-, and 5-year OS and CSS prediction models, shown as nomograms (**Figures 3A, B**).

**Table 2:** Multivariate Cox regression analysis of the OS and CSS in development set.

Variables	Comparison groups	Overall survival			Cancer-specific survival		
		β	HR, 95% CI	*P* value	β	HR, 95% CI	*P* value
Tumor grade	III–IV vs. I–II	0.48	1.62 (1.38, 1.90)	< 0.001	0.46	1.59 (1.35, 1.88)	< 0.001
AJCC T stage	T4 vs. T1–T3	0.30	1.35 (1.17, 1.56)	< 0.001	0.31	1.36 (1.17, 1.58)	< 0.001
AJCC N stage	N2 vs. N0–N1	0.26	1.30 (1.12, 1.50)	< 0.001	0.32	1.38 (1.18, 1.61)	< 0.001
Chemotherapy	Yes vs. no	-1.01	0.37 (0.31, 0.43)	< 0.001	-0.98	0.38 (0.32, 0.44)	< 0.001
CEA	Positive vs. negative	0.25	1.28 (1.06, 1.55)	0.011	0.26	1.30 (1.06, 1.59)	0.010
Liver metastasis	Yes vs. no	0.58	1.79 (1.53, 2.09)	< 0.001	0.60	1.82 (1.55, 2.15)	< 0.001

β, coefficient; HR, hazard ratio.

### Validation of prediction models

**Figure 4:**
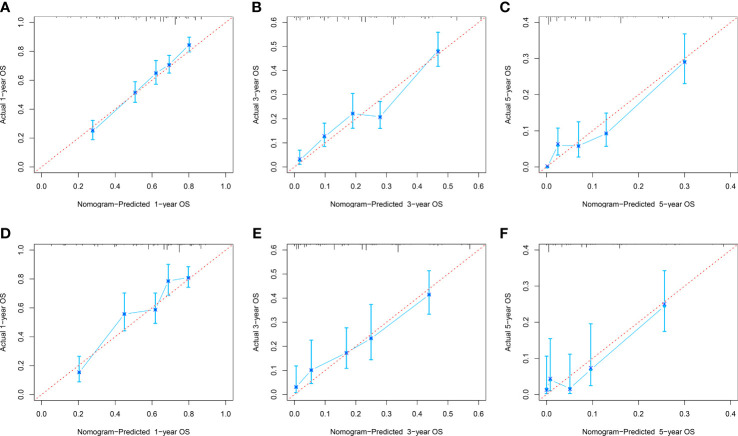
Calibration curves of 1-, 3-, and 5-year OS in the development **(A–C)** and validation **(D–E)** sets.

**Figure 5:**
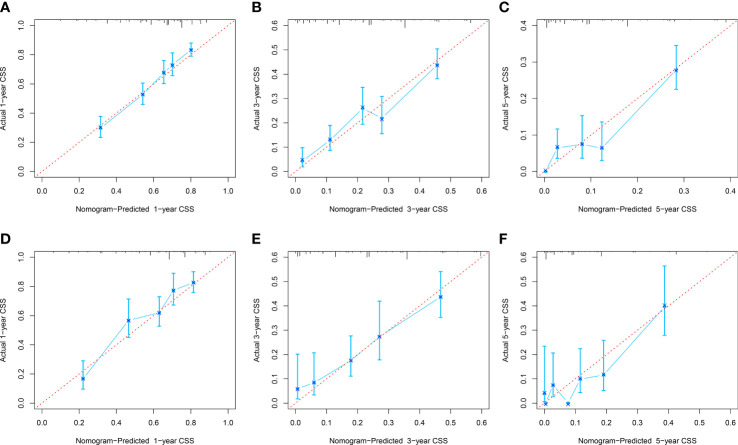
Calibration curves of 1-, 3-, and 5-year CSS in the development **(A–C)** and validation **(D–F)** sets.

**Figure 6:**
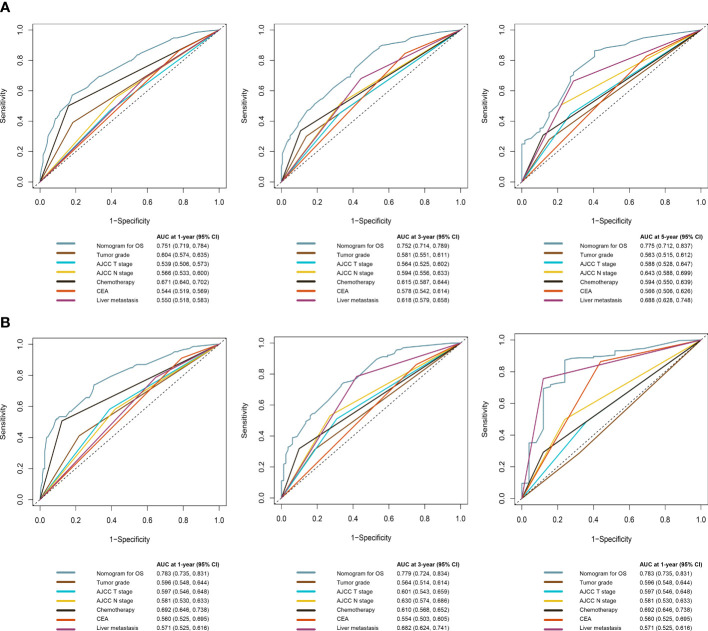
Time-dependent ROC curves comparing the prognostic accuracy of the OS prediction model with clinical risk factors in the development **(A)** and validation **(B)** sets.

**Figure 7:**
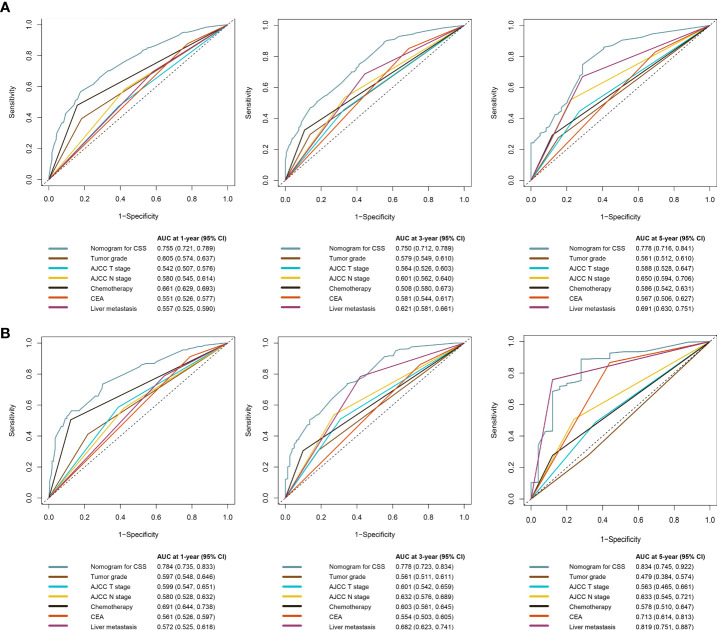
Time-dependent ROC curves comparing the prognostic accuracy of the CSS prediction model with clinical risk factors in development **(A)** and validation **(B)** sets.

**Figure 8:**
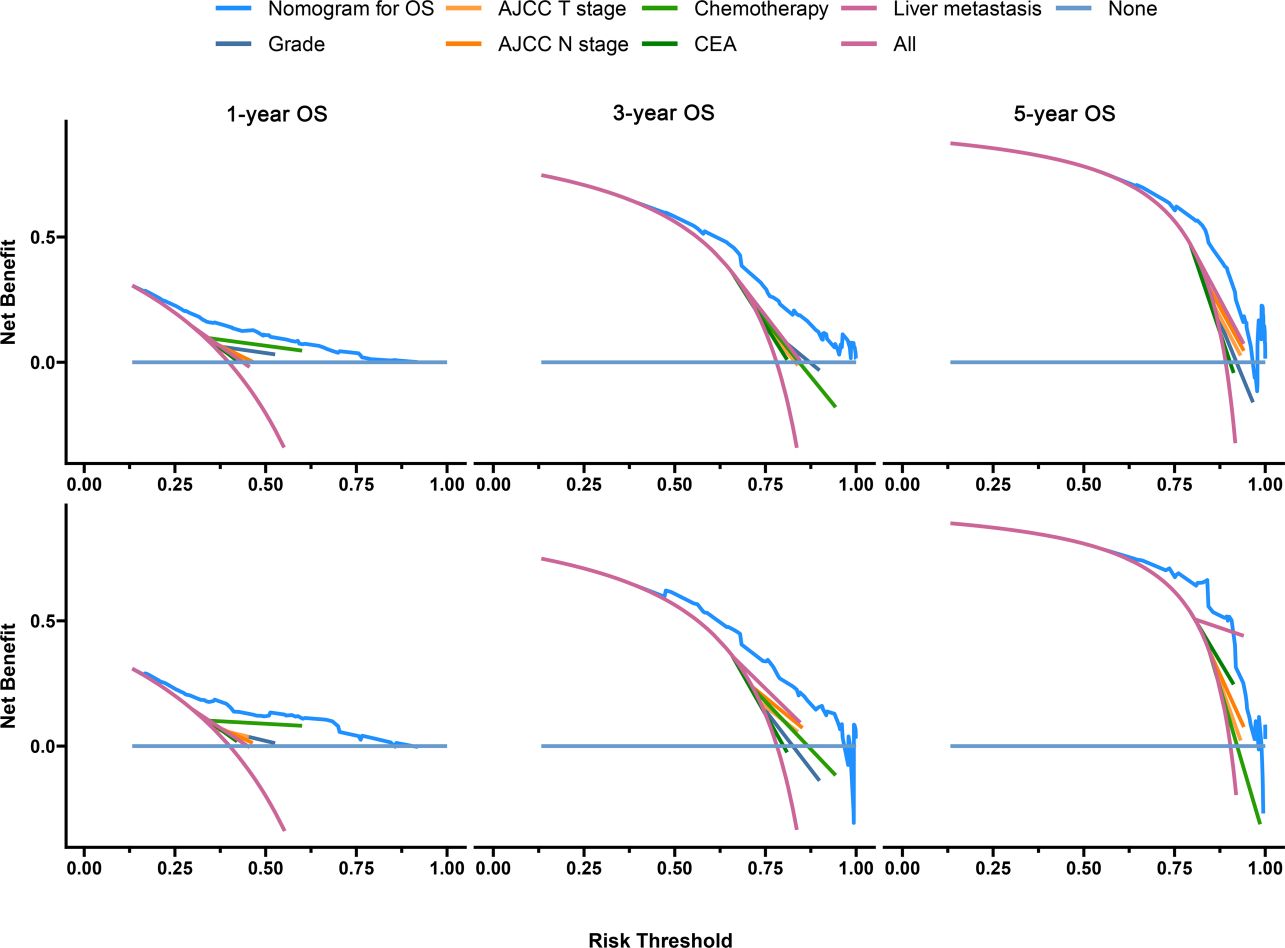
Decision curves of the OS prediction model in the development (upper) and validation (lower) sets.

**Figure 9:**
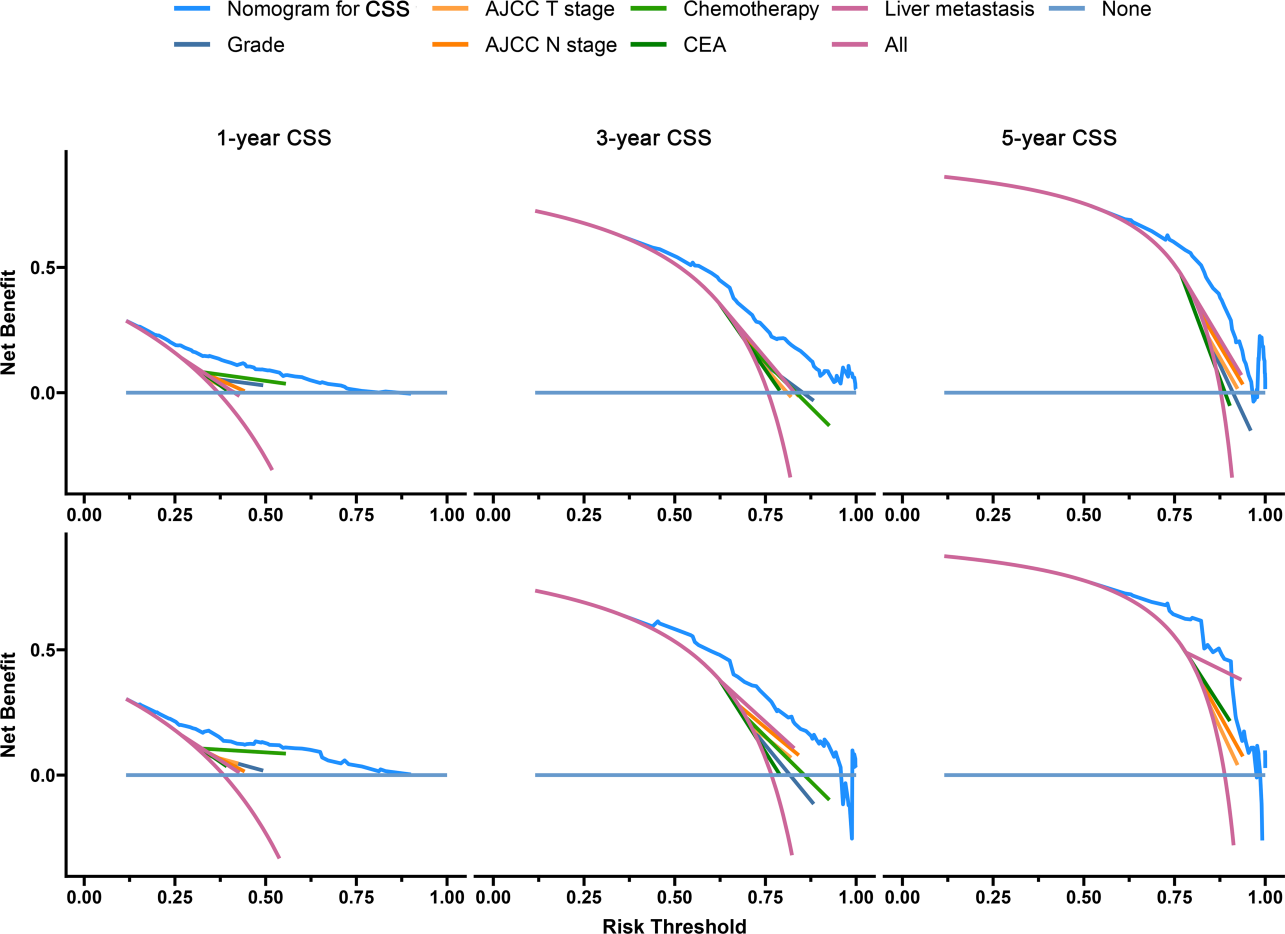
Decision curves of the CSS prediction model in the development (upper) and validation (lower) sets.

The C-index of the OS prediction model was 0.685 (95% CI, 0.664–0.705) in the development set and 0.716 (95% CI, 0.686–0.745) in the validation set. Meanwhile, the C-index of the CSS prediction model was 0.688 (95% CI, 0.666–0.710) in the development set and 0.713 (95% CI, 0.682–0.743) in the validation set. The calibration curve plots of two prognosis prediction models revealed an excellent agreement between the predicted and actual risks (**Figures 4**, **5**). The time-dependent ROC curves were used to compare the predictive performance of each prognostic factor and prediction model, which showed higher predictability of two models than any independent factor, with the 1-, 3-, and 5-year AUCs for the OS prediction model of 0.751 (95% CI, 0.719–0.784), 0.752 (95% CI, 0.714–0.789), and 0.775 (95% CI, 0.712–0.837) in the development set (**Figure 6A**) and 0.783 (95% CI, 0.735–0.831), 0.779 (95% CI, 0.724–0.834), and 0.834 (95% CI, 0.743–0.924) in the validation set (**Figure 6B**). Equally, the ROC curves for the CSS prediction model revealed satisfactory results, with the 1-, 3-, and 5-year AUCs of 0.755 (95% CI, 0.721–0.789), 0.750 (95% CI, 0.712–0.789), and 0.778 (95% CI, 0.716–0.841) in the development set (**Figure 7A**) and 0.784 (95% CI, 0.735–0.833), 0.778 (95% CI, 0.723–0.834), and 0.834 (95% CI, 0.745–0.922) in the validation set (**Figure 7B**). Furthermore, the results of DCA also indicated better clinical applicability of two prediction models than any single risk factor (**Figures 8**, **9**).

### Survival analysis

**Figure 10:**
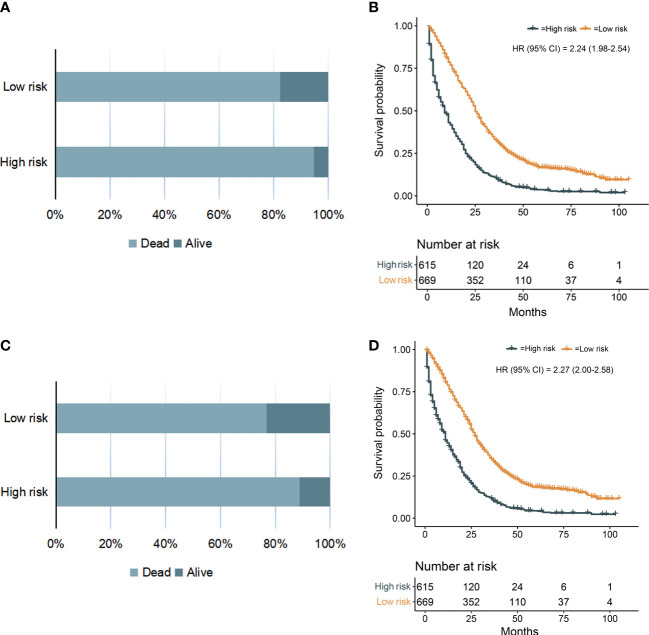
The OS **(A)** and CSS **(C)** status of CCLM patients in the low-risk and high-risk groups. Kaplan–Meier curves for OS **(B)** and CSS **(D)** of all 1,284 cases with CCLM in the low-risk and high-risk groups.

**Figure 11:**
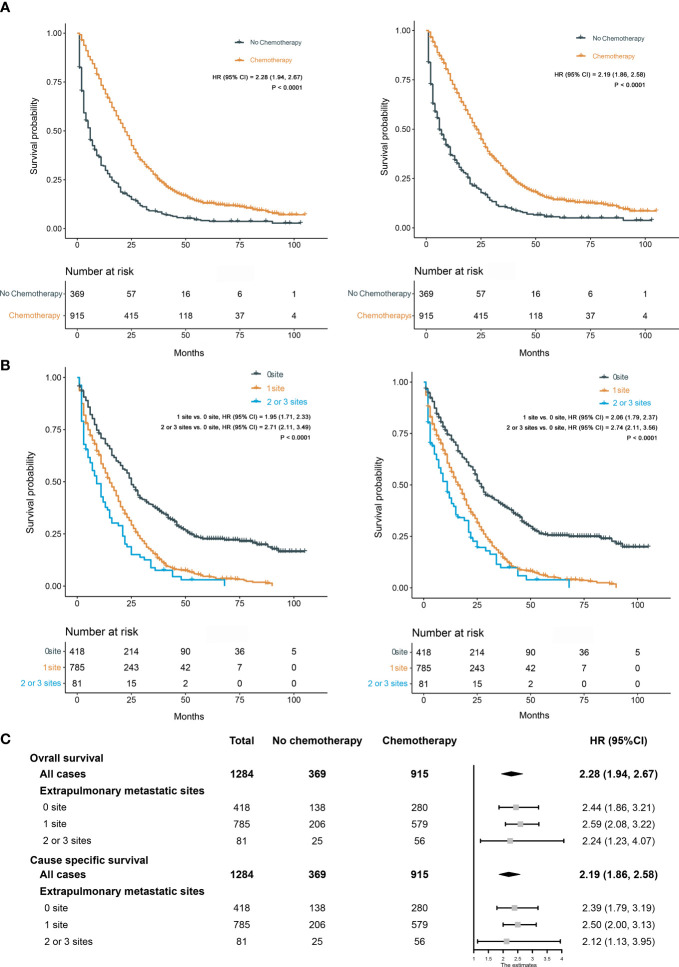
**(A)** Kaplan–Meier curves for OS (left) and CSS (right) between chemotherapy and no chemotherapy. **(B)** Kaplan–Meier curves for OS (left) and CSS (right) among different extents of extrapulmonary metastases. **(C)** Effect of chemotherapy on OS and CSS in different subgroups stratified by the extent of extrapulmonary metastases.

We calculated the risk score for all patients according to the constructed prediction models, and the patients were divided into low-risk and high-risk groups according to the median risk score. The statistical analysis for all-cause and cancer-specific mortality revealed higher levels in the high-risk group than in the low-risk group (all-cause mortality: low-risk vs. high risk, 82.4% vs. 94.8%, *P* < 0.0001; cancer-specific mortality: low-risk vs. high risk, 76.7% vs. 88.9%, *P* < 0.0001; **Figures 10A, C**). **Supplementary Table S2** shows the statistics of survival status at the end of follow-up, which revealed that the high-risk group had a higher mortality in almost each subgroup stratified by tumor grade, AJCC T stage, AJCC N stage, chemotherapy, CEA, and liver metastasis. The K-M plots and log-rank test also showed the worse OS and CSS conditions in the high-risk group than in the low-risk group (*P* < 0.0001, **Figures 10B, D**); furthermore, we conducted subgroup K-M analysis between low-risk and high-risk groups after stratifying by risk factors, still indicating a worse survival in the high-risk group (**Supplementary Table S3**). We noted that the overall cohort had a favorable response to chemotherapy (OS, HR = 2.24; 95% CI, 1.98–2.54; CSS, HR = 2.27; 95% CI, 2.00–2.58; **Figure 11A**). Survival analysis showed that more extrapulmonary metastatic sites indicated poorer prognosis, graphically displayed in **Figure 11B**. Based on the extent of extrapulmonary metastases, we performed the subgroup analysis to evaluate the enhanced effect of chemotherapy in survival. Among CCLM patients with or without extrapulmonary metastases, chemotherapy could provide a good prognostic opportunity (**Figure 11C**).

### Expanded validation of prediction models

We redownloaded the data of patients with complete tumor grade, AJCC T stage, AJCC N stage, chemotherapy, CEA, and liver metastasis information from the SEER database as an expanded testing set (n = 3115; **Supplementary Table S4**). The C-indexes of OS and CSS prediction models were 0.671 (95% CI, 0.665–0.677) and 0.672 (95% CI, 0.666–0.678), respectively. Calibration plots for two models showed good consistency between the predicted and actual risks (**Supplementary Figure S1**). The time-dependent ROC curves of two models were over the curves of each single prognostic factor (**Supplementary Figure S2**). Also, DCA still indicated that two models could yield more ideal clinical benefits than a single prognostic factor (**Supplementary Figure S3**). The statistics of survival status showed different all-cause and cancer-specific mortalities between high-risk and low-risk groups (*P* < 0.0001), and survival analysis also suggested the difference of survival patterns between these two groups (*P* < 0.0001), as shown in **Supplementary Figure S4**.

## Discussion

Previous studies investigated prognostic factors and constructed prediction models for colon cancer with distant organ metastases (5, 6, 19). However, the study on the establishment of prognostic models for CCLM was lacking. To explore the prognosis of the unique CCLM subset patients, we performed the identification of prognostic risk factors and development of prediction models in this study to provide valuable guidance for clinical prognosis estimation and individualized administration of patients with CCLM.

In this study, we established prediction models for the 1-, 3-, and 5-year prognosis of CCLM based on a mass of clinical samples from the SEER database. Six parameters (i.e., tumor grade, AJCC T stage, AJCC N stage, chemotherapy, CEA, liver metastasis), significantly associated with the OS and CSS of CCLM patients, were incorporated as independent prognostic factors. The analysis showed that patients in tumor grades III–IV had a 62% increased risk of overall death (HR, 1.62) and a nearly 60% increased risk of cancer-specific death (HR, 1.59), in comparison with patients in I–II tumor grades. According to the results of LASSO regression analysis, we excluded the parameter “tumor size,” which probably has certain collinearity with “AJCC T stage.” Furthermore, lymph node metastasis was the common form of CC metastasis. Previous studies had proved that the higher N stage indicated the worse prognosis in metastatic CC patients (20, 21). Similarly, CCLM patients in the N2 stage had worse prognosis than in the N0–N1 stages based on our analysis. Population and evidence had shown that chemotherapy could provide metastasis CC patients with survival advantage (22, 23). On the strength of our analysis, chemotherapy was also considered to be critical for improving CCLM patients’ prognosis; furthermore, it could be conducive to significantly enhance survival in the subgroup population of CCLM with extrapulmonary metastases. However, chemotherapeutics could produce additional toxicity (e.g., neurotoxicity) to bring about a lot of adverse effects for patients; not all patients would derive good benefit. Thus, consideration of each patient’s specific clinical fact was important in decision of using adjuvant therapy for CC (22, 24, 25). Meanwhile, Liu et al. mentioned that a study reported that CC patients could gain benefits through adjuvant radical treatment; however, “radiotherapy” was excluded based on LASSO regression analysis (26). Probably, also as an adjunctive therapy, “radiotherapy” may have collinearity with “chemotherapy” (27). In our study, CEA positive was also a prognostic risk factor in CCLM patients. The elevated preoperative level of CEA had an intimate relationship with bad tumor stage and impaired the patient’s surgical benefit profile, leading to the shortened 5-year survival rates (28, 29). The return to normal levels of serum CEA after lung metastasectomy usually indicated a better prognosis; hence, close monitoring of CEA levels was quite important for the postoperative management of patients. As the most common form of distant metastasis in CC, liver metastases can increase the risk of death in patients (26). Similarly, liver metastasis was identified as a risk factor for CCLM in our study; however, bone metastasis and brain metastasis were not identified as risk factors, which may be due to the low proportion of patients with bone or brain metastases in the current cohort. In addition, 67.4% of CC patients with pulmonary metastases at diagnosis will have other simultaneous metastatic sites to the liver, bone, and brain. Hence, it is essential to screen for these sites in the clinic.

Based on the results of the model validation, all the C-indexes and AUCs were more than or close to 0.7 and the proximity of calibration curves for models to the diagonal was excellent, indicating that the models had excellent predictability and accuracy. All the ROC curves of single prognostic factors were under curves of the constructed prediction models, which showed that models had better predictability than any independent risk factor. Furthermore, DCA was recommended in many leading medical journals including *Lancet Oncology*, *BMJ*, and *PLOS Medicine* (30). Therefore, DCA was performed in this study, and the results indicated that prediction models could yield higher clinical benefits than any single risk factor. Of note, there were significant differences in the primary site distribution and liver metastasis between the development and validation sets. This might be the reason why the predictability, accuracy, and clinical benefits of models in the validation set are slightly worse than those in the development set but still were satisfactory. On the risk scores calculated by prediction models, we divided the entire cohort into low-risk and high-risk groups. In the entire cohort, the all-cause and cancer-specific mortalities of patients in the high-risk group were 12.4% and 12.2% more than in the low-risk group, respectively. Most of subgroup analyses after stratifying by risk factor also suggested that the mortality in the high-risk group was higher than in the low-risk group. Additionally, the K-M survival analysis of all cases indicated that the low-risk group had a better prognosis than the high-risk group. Stratified by different risk factors, the prognosis of patients between the two groups was compared by subgroup analyses. The results still did not change, suggesting that models had significant discrimination for patients with different risks in the 1-, 3-, and 5-year prognoses. Furthermore, we collected an expanded testing set with 3,115 CCLM patients from the SEER database and model validation in this set still showed satisfactory results.

Nomogram was used to show and apply our prediction models as a convenient form to predict various clinical outcomes, providing better guidance for CCLM-individualized medical judgement and decision-making (31). Also, all the prognostic factors identified in our study were easily available in clinical practice, allowing for the more convenient operation and application. Currently, Huang et al., Li et al., and Wang et al. have developed similar prognostic models, focusing on the overall cohort of CRC or the cohort of CCLM without liver metastasis (32–34); however, our models are specific for the prognostic evaluation of unique CCLM subset patients. Meanwhile, we categorized the SEER database data as a development set and a validation set using stratified randomization, which could prevent imbalance between two groups for known factors that influence prognosis. Unlike prior studies, model validation was performed using the independent validation dataset that was not applied to train models, allowing for more effective testing of the performance of the model.

There were still some shortcomings in the present study. First, a mass of data with missing or unclear information were excluded, exacerbating the risk of selection bias. Surgery was a recognized protective factor, and the resection of the primary and metastatic lesions in patients with metastatic CC results in 5-year survival rates of 20%–50% (35). However, almost all patients (99.8%) underwent surgery in the present study. We did not evaluate the association between survival and whether surgery was performed, without obtaining the patients’ data with surgery difference from the SEER database. This was probably because the data were excluded in the data preprocessing. Further data and studies were needed to explore this point and cover the current shortage. Second, some important information was not recorded in the SEER database, including secondary tumor size, number, and depth of invasion of the metastatic lesions, metastasis to one lung or both lungs, and specific chemotherapy regimens. Third, today is the era of precision medicine; simple clinical and pathological characteristics in this study may not satisfy the evaluation of prognosis for tumors. Integrating multiple biomarkers with clinical characteristics may provide a more substantial prognostic value (36). However, in the present study, we were unable to conduct this analysis due to the lack of relevant data in SEER. Fourth, the study was a retrospective rather than a prospective cohort study; recall bias was inherent in retrospective studies. Fifth, the patients in the present study got diagnosed between 2010 and 2015. With recent medical advances, many new therapies have been applied to cancer patients. The application of immunotherapy and new chemotherapeutic drugs has changed the prognosis of patients to a certain degree. Hence, the clinical guidance that can be given by this study was limited. In the future, more new studies are needed to get more excellent prediction models.

## Conclusion

Based on the clinical variables in the SEER database, we constructed and validated the prediction models for 1-, 3-, and 5-year OS and CSS of patients with CCLM. The prognosis prediction models could provide effective clinical prognostic evaluation for patients with CCLM and guide clinicians to optimize individualized treatment.

## Data availability statement

Publicly available datasets were analyzed in this study. The datasets analyzed in this study are available in the SEER repository and can be obtained from: https://seer.cancer.gov/.

## Author contributions

HY and ZM conceived and designed the study. ZM, SY, YY, and JL carried out the collection and preprocessing of data. JL, ZM, and YZ processed the figures and tables. ZM, SY, YY, and YZ undertook the statistical analysis. ZM and SY drafted the manuscript. HY, ZM, and YY participated in manuscript revision. All authors contributed to the article and approved the submitted version.
