# Supplementary material for: Development and validation of prediction models for the prognosis of colon cancer with lung metastases: a population-based cohort study
Source: Front Endocrinol (Lausanne). 2023 Jul 31;14:1073360. doi: 10.3389/fendo.2023.1073360 (PMC10424923; doi:10.3389/fendo.2023.1073360)
Supplement: Supplementary file 1 [file DataSheet_1.pdf]

# Development and validation of prediction models for the prognosis of colon cancer with lung metastases: A population-based cohort study

**Zhenyu Ma[1], Shuping Yang[1], Yalin Yang[1], Jingran Luo[1], Yixiao Zhou[1], Huiyong Yang[1*]**

[1]School of Medicine, Huaqiao University, Quanzhou 362021, China

*** Correspondence:**

Corresponding author: Huiyong Yang

E-mail: baiqingyang_78@163.com

**Table S1.** Comparison of clinicopathological features and treatment options between cohorts CCLM without extrapulmonary metastases and CCLM with extrapulmonary metastases.

| Variable | Overall patients (*N* = 1284) | CCLM without extrapulmonary metastases (N = 418) | CCLM with extrapulmonary metastases (N = 866) | χ2 | *P* value |
|---|---|---|---|---|---|
| **Age** | | | | | |
| < 65 | 635 (49.5%) | 194 (46.4%) | 441 (50.9%) | 2.12 | 0.15 |
| ≥ 65 | 649 (50.5%) | 224 (53.6%) | 425 (49.1%) | | |
| **Sex** | | | | | |
| Female | 628 (48.9%) | 219 (52.4%) | 409 (47.2%) | 2.81 | 0.09 |
| Male | 656 (51.1%) | 199 (47.6%) | 457 (52.8%) | | |
| **Race** | | | | | |
| White | 922 (71.8%) | 290 (69.4%) | 632 (73.0%) | 1.82 | 0.40 |
| Black | 219 (17.1%) | 77 (18.4%) | 142 (16.4%) | | |
| Others* | 143 (11.1%) | 51 (12.2%) | 92 (10.6%) | | |
| **Primary site** | | | | | |
| Ascending colon | 224 (17.4%) | 75 (17.9%) | 149 (17.2%) | 3.86 | 0.43 |
| Transverse colon | 117 (9.1%) | 41 (9.8%) | 76 (8.8%) | | |
| Descending colon | 85 (6.6%) | 34 (8.1%) | 51 (5.9%) | | |
| Sigmoid colon | 415 (32.3%) | 135 (32.3%) | 280 (32.3%) | | |
| Others** | 443 (34.5%) | 133 (31.8%) | 310 (35.8%) | | |
| **Tumor grade** | | | | | |
| I-II | 939 (73.1%) | 308 (73.7%) | 631 (72.9%) | 0.06 | 0.81 |
| III-IV | 345 (26.9%) | 110 (26.3%) | 235 (27.1%) | | |
| **AJCC T stage** | | | | | |
| T1-T3 | 725 (56.5%) | 252 (60.3%) | 473 (54.6%) | 3.46 | 0.06 |
| T4 | 559 (43.5%) | 166 (39.7%) | 393 (45.4%) | | |
| **AJCC N stage** | | | | | |
| N0-N1 | 672 (52.3%) | 255 (61.0%) | 417 (48.2%) | 18.16 | < 0.01 |
| N2 | 612 (47.7%) | 163 (39.0%) | 449 (51.8%) | | |
| **Radiotherapy** | | | | | |
| No/unknown | 1209 (94.2%) | 405 (96.9%) | 804 (92.8%) | 7.68 | < 0.01 |
| Yes | 75 (5.8%) | 13 (3.1%) | 62 (7.2%) | | |
| **Chemotherapy** | | | | | |
| No/unknown | 369 (28.7%) | 138 (33.0%) | 231 (26.7%) | 5.23 | 0.02 |
| Yes | 915 (71.3%) | 280 (67.0%) | 635 (73.3%) | | |
| **CEA** | | | | | |
| Negative | 232 (18.1%) | 140 (33.5%) | 92 (10.6%) | 98.06 | < 0.01 |
| Positive | 1052 (81.9%) | 278 (66.5%) | 774 (89.4%) | | |
| **Marital status** | | | | | |
| Married | 697 (54.3%) | 210 (50.2%) | 487 (56.2%) | 3.85 | 0.05 |
| Unmarried | 587 (45.7%) | 208 (49.8%) | 379 (43.8%) | | |

| | | | | | |
|---|---|---|---|---|---|
| **Tumor size** | | | | | |
| < 5cm | 520 (40.5%) | 177 (42.3%) | 343 (39.6%) | 1.98 | 0.37 |
| 5-10cm | 117 (9.1%) | 209 (50.0%) | 438 (50.6%) | | |
| > 10cm | 647 (50.4%) | 32 (7.7%) | 85 (9.8%) | | |
| **Bone metastasis** | | | | | |
| No | 1197 (93.2%) | 418 (100%) | 779 (90.0%) | NA | NA |
| Yes | 87 (6.8%) | 0 (0%) | 87 (10.0%) | | |
| **Brain metastasis** | | | | | |
| No | 1256 (97.8%) | 418 (100%) | 838 (96.8%) | NA | NA |
| Yes | 28 (2.2%) | 0 (0%) | 28 (3.2%) | | |
| **Liver metastasis** | | | | | |
| No | 449 (35.0%) | 418 (100%) | 31 (3.6%) | NA | NA |
| Yes | 835 (65.0%) | 0 (0%) | 835 (96.4%) | | |
| **Tumor number** | | | | | |
| Single | 1025 (79.8%) | 322 (77.0%) | 703 (81.2%) | 2.76 | 0.10 |
| Multiple | 259 (20.2%) | 96 (23.0%) | 163 (18.8%) | | |
| **Surgery sites** | | | | | |
| Only primary site | 1026 (79.9%) | 354 (84.7%) | 672 (77.6%) | 8.93 | 0.01 |
| Primary & other sites† | 255 (19.9%) | 63 (15.1%) | 192 (22.2%) | | |
| No surgery | 3 (0.2%) | 1 (0.2%) | 2 (0.2%) | | |
| **Histological type** | | | | | |
| Adenocarcinoma | 1170 (91.1%) | 374 (89.5%) | 796 (91.9%) | 1.79 | 0.18 |
| Others‡ | 114 (8.9%) | 44 (10.5%) | 70 (8.1%) | | |

*Asian or Pacific Islander, American Indian/Alaska Native; ** Cecum, appendix, hepatic flexure of colon, splenic flexure of colon, and overlapping lesion of colon; † Surgery of other sites describes the surgical removal of distant lymph nodes or other tissues or organs beyond the primary site; ‡ Cystic, mucinous and serous neoplasms, ductal and lobular neoplasmsm, complex epithelial neoplasms; CEA = carcino-embryonic antigen.

**Table S2**. Summary of the OS and CSS status stratified by different risk factors in all 1284 patients.

| Varibales | Entire cohort, No. | Overall survival | | | | | Cancer-specific survival | | | | |
|---|---|---|---|---|---|---|---|---|---|---|---|
| | | Low-risk | | High-risk | | | Low-risk | | High-risk | | |
| | | Patients, No. | Survival status | Patients, No. | Survival status | *P* value | Patients, No. | Survival status | Patients, No. | Survival status | *P* value |
| I-II | 939 | 605 | Alive: 107 (17.7%)<br>Dead: 498 (82.3%) | 334 | Alive: 18 (5.4%)<br>Dead: 316 (94.6%) | < 0.0001 | 605 | Alive: 143 (23.6%)<br>Dead: 462 (76.4%) | 334 | Alive: 34 (10.2%)<br>Dead: 300 (89.8%) | < 0.0001 |
| III-IV | 345 | 64 | Alive:11 (17.2%)<br>Dead: 53 (82.8%) | 281 | Alive: 14 (5.0%)<br>Dead: 267 (95.0%) | 0.0017 | 64 | Alive: 13 (20.3%)<br>Dead: 51 (79.7%) | 281 | Alive: 34 (12.1%)<br>Dead: 247 (87.9%) | 0.1268 |
| T1-T3 | 725 | 464 | Alive: 90 (19.4%)<br>Dead: 374 (80.6%) | 261 | Alive: 21 (8.0%)<br>Dead: 240 (92.0%) | < 0.0001 | 464 | Alive: 116 (25.0%)<br>Dead: 348 (75.0%) | 261 | Alive: 39 (14.9%)<br>Dead: 222 (85.1%) | 0.0021 |
| T4 | 559 | 205 | Alive: 28 (13.7%)<br>Dead: 177 (86.3%) | 354 | Alive: 11 (3.1%)<br>Dead: 343 (96.9%) | < 0.0001 | 205 | Alive: 40 (19.5%)<br>Dead: 165 (80.5%) | 354 | Alive: 29 (8.2%)<br>Dead: 325 (91.8%) | 0.0002 |
| N0-N1 | 672 | 458 | Alive: 93 (20.3%)<br>Dead: 365 (79.7%) | 214 | Alive: 16 (7.5%)<br>Dead: 198 (92.5%) | < 0.0001 | 458 | Alive: 124 (27.1%)<br>Dead: 334 (72.9%) | 214 | Alive: 36 (16.8%)<br>Dead: 178 (83.2%) | 0.0050 |
| N2 | 612 | 211 | Alive: 25 (11.8%)<br>Dead: 186 (88.2%) | 401 | Alive: 16 (4.0%)<br>Dead: 385 (96.0%) | 0.0004 | 211 | Alive: 32 (15.2%)<br>Dead: 179 (84.8%) | 401 | Alive: 32 (8.0%)<br>Dead: 369 (92.0%) | 0.0087 |
| No/unknown | 369 | 16 | Alive: 4 (25.0%)<br>Dead: 12 (75.0%) | 353 | Alive:20 (5.7%)<br>Dead: 333 (94.3%) | 0.0108 | 16 | Alive: 9 (56.3%)<br>Dead: 7 (43.7%) | 353 | Alive: 47 (13.3%)<br>Dead: 306 (86.7%) | < 0.0001 |
| Yes | 915 | 653 | Alive:114 (17.5%)<br>Dead: 539 (82.5%) | 262 | Alive: 12 (4.6%)<br>Dead: 250 (95.4%) | < 0.0001 | 653 | Alive: 147 (22.5%)<br>Dead: 506 (77.5%) | 262 | Alive: 21 (8.0%)<br>Dead: 241 (92.0%) | < 0.0001 |
| Negative | 232 | 153 | Alive:42 (27.5%)<br>Dead: 111 (72.5%) | 79 | Alive: 7 (8.9%)<br>Dead: 72 (91.1%) | 0.0018 | 153 | Alive: 53 (34.6%)<br>Dead: 100 (65.4%) | 79 | Alive: 12 (15.2%)<br>Dead: 67 (84.8%) | 0.0030 |
| Positive | 1052 | 516 | Alive: 76 (14.7%)<br>Dead: 440 (85.3%) | 536 | Alive: 25 (4.7%)<br>Dead: 511 (95.3%) | < 0.0001 | 516 | Alive: 103 (20.0%)<br>Dead: 413 (80.0%) | 536 | Alive: 56 (10.4%)<br>Dead: 480 (89.6%) | < 0.0001 |

| | | | | | | | | | | | |
|---|---|---|---|---|---|---|---|---|---|---|---|
| No | 449 | 298 | Alive: 87 (29.2%)<br>Dead: 211 (70.8%) | 151 | Alive: 15 (9.9%)<br>Dead: 136 (90.1%) | < 0.0001 | 298 | Alive: 107 (35.9%)<br>Dead: 191 (64.1%) | 151 | Alive: 25 (16.6%)<br>Dead: 126 (83.4%) | < 0.0001 |
| Yes | 835 | 371 | Alive: 31 (8.4%)<br>Dead: 340 (91.6%) | 464 | Alive: 17 (3.7%)<br>Dead: 447 (96.3%) | 0.0061 | 371 | Alive: 49 (13.2%)<br>Dead: 322 (86.8%) | 464 | Alive: 43 (9.3%)<br>Dead: 421 (90.7%) | 0.0900 |

**Table S3**. The results of survival analysis for all 1284 cases with CCLM stratified by different risk factors in low-risk and high-risk groups.

| **Subgroups** | **Overall survival** | | **Cancer-specific survival** | |
|---|---|---|---|---|
| | **HR (95% CI)** | ***P* value** | **HR (95% CI)** | ***P* value** |
| Grade (I-II) | 2.22 (1.88, 2.62) | < 0.0001 | 2.28 (1.92, 2.71) | < 0.0001 |
| Grade (III-IV) | 2.06 (1.62, 2.63) | < 0.0001 | 1.99 (1.55, 2.55) | < 0.0001 |
| AJCC T stage (T1-T3) | 2.20 (1.62, 2.63) | < 0.0001 | 2.21 (1.81, 2.69) | < 0.0001 |
| AJCC T stage (T4) | 2.11 (1.78, 2.51) | < 0.0001 | 2.16 (1.81, 2.58) | < 0.0001 |
| AJCC N stage (N0-N1) | 2.34 (1.89, 2.89) | < 0.0001 | 2.32 (1.86, 2.89) | < 0.0001 |
| AJCC N stage (N2) | 1.94 (1.65, 2.29) | < 0.0001 | 1.94 (1.64, 2.30) | < 0.0001 |
| Chemotherapy (No/unknown) | 2.14 (1.42, 3.22) | 0.0051 | 3.39 (2.21, 5.20) | 0.00035 |
| Chemotherapy (Yes) | 1.92 (1.61, 2.30) | < 0.0001 | 1.98 (1.65, 2.37) | < 0.0001 |
| CEA (Negative) | 2.36 (1.66, 3.36) | < 0.0001 | 2.49 (1.72, 3.60) | < 0.0001 |
| CEA (Positive) | 2.15 (1.88, 2.45) | < 0.0001 | 2.15 (1.88, 2.47) | < 0.0001 |
| Liver metastasis (No) | 2.59 (1.99, 3.37) | < 0.0001 | 2.68 (2.03, 3.54) | < 0.0001 |
| Liver metastasis (Yes) | 1.87 (1.63, 2.16) | < 0.0001 | 1.87 (1.62, 2.16) | < 0.0001 |

**Table S4**. Baseline characteristics of all 3115 patients in expanded testing set.

| Variable | Overall patients (N = 3115) |
| --- | --- |
| **Tumor grade** | |
| I-II | 2398 (77.0%) |
| III-IV | 717 (23.0%) |
| **AJCC T stage** | |
| T1-T3 | 2070 (66.5%) |
| T4 | 1045 (33.5%) |
| **AJCC N stage** | |
| N0-N1 | 2167 (69.6%) |
| N2 | 948 (30.4%) |
| **Chemotherapy** | |
| No/unknown | 730 (23.4%) |
| Yes | 2385 (76.6%) |
| **CEA** | |
| Negative | 548 (17.6%) |
| Positive | 2567 (82.4%) |
| **Liver metastasis** | |
| No | 1055 (33.9%) |
| Yes | 2060 (66.1%) |
| **Overall survival status** | |
| Alive | 345 (11.1%) |
| Dead | 2770 (88.9%) |
| **Cancer-specific survival status** | |
| Alive | 519 (16.7%) |
| Dead | 2596 (83.3%) |
| **Survival months** | |
| Median (interquartile range [IQR]) | 17 (6-32) |

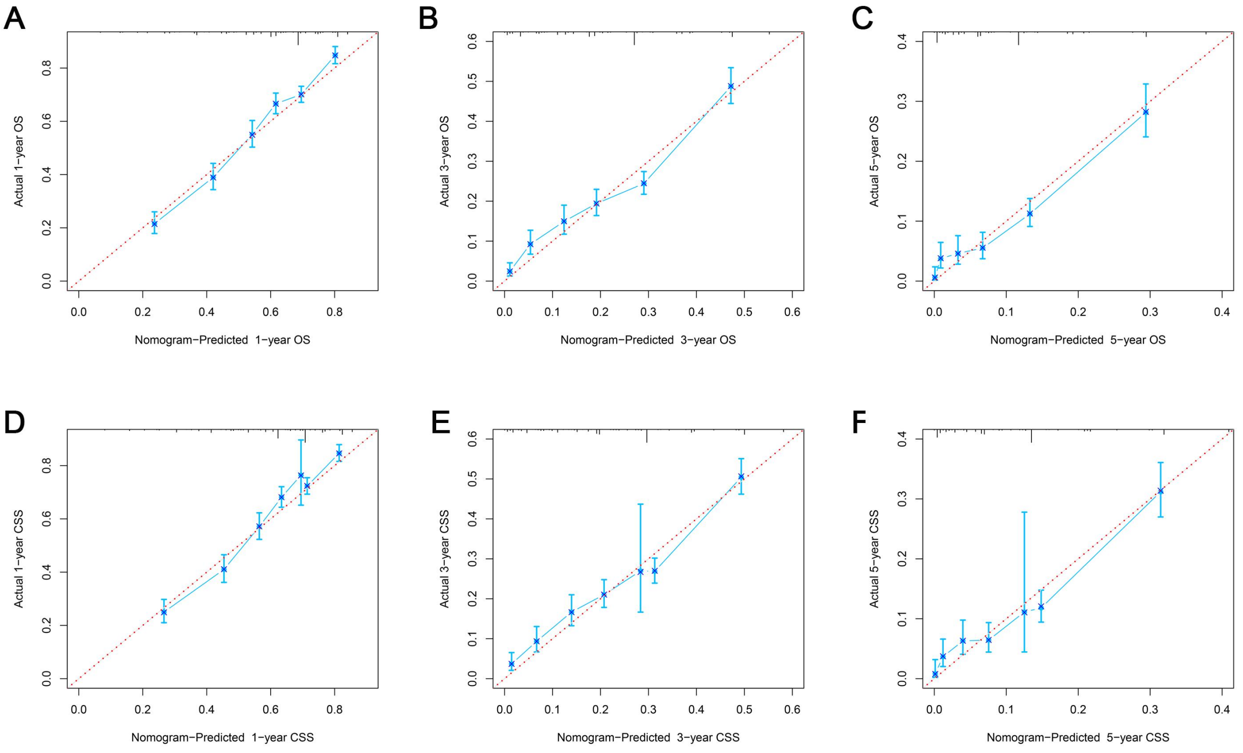


**Figure S1**. Calibration curves of 1-, 3-, 5-year OS (A, B, C) and CSS (D, E, F) in expanded testing set.

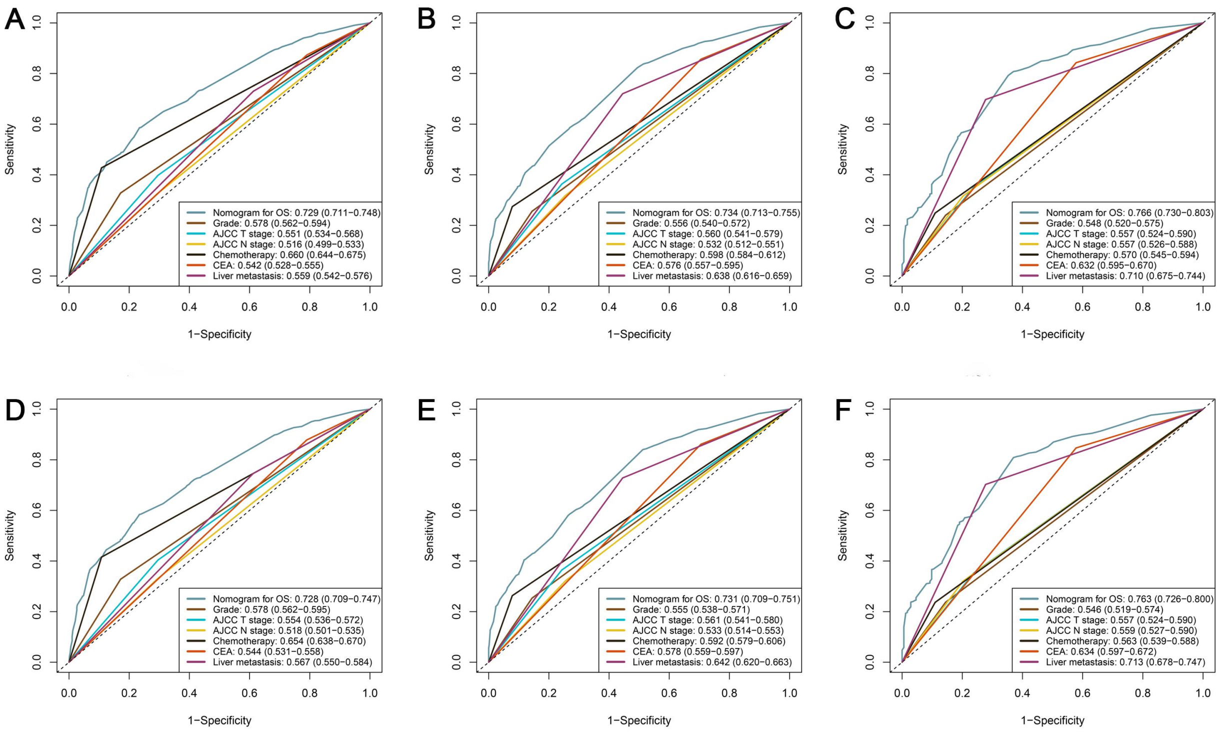


**Figure S2**. Time-dependent ROC curves comparing the prognostic accuracy of the 1-, 3-, 5-year OS (A, B, C) and CSS (D, E, F) prediction model with clinical risk factors in expanded testing set.

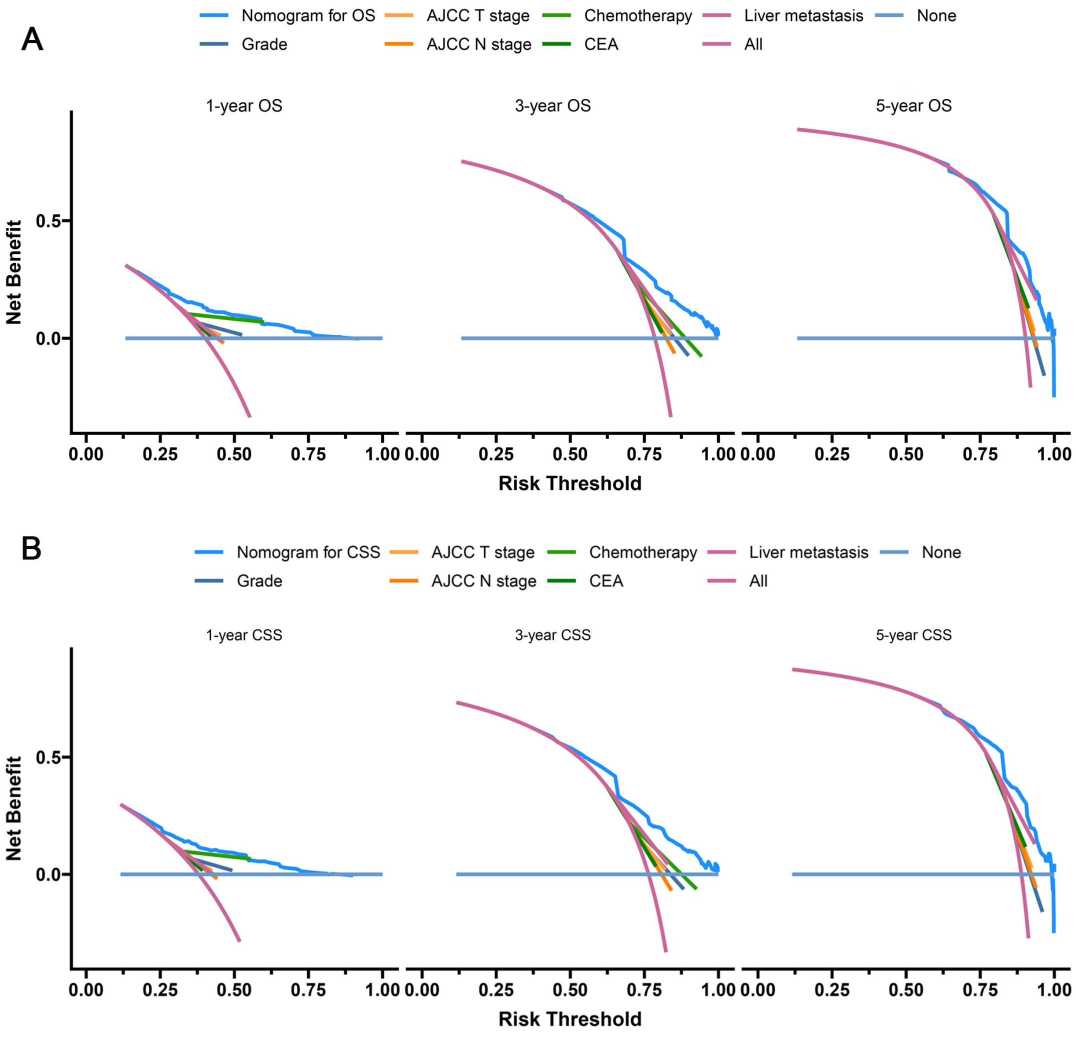


**Figure S3**. Decision curves of the OS (A) and CSS (B) prediction model in expanded testing set.

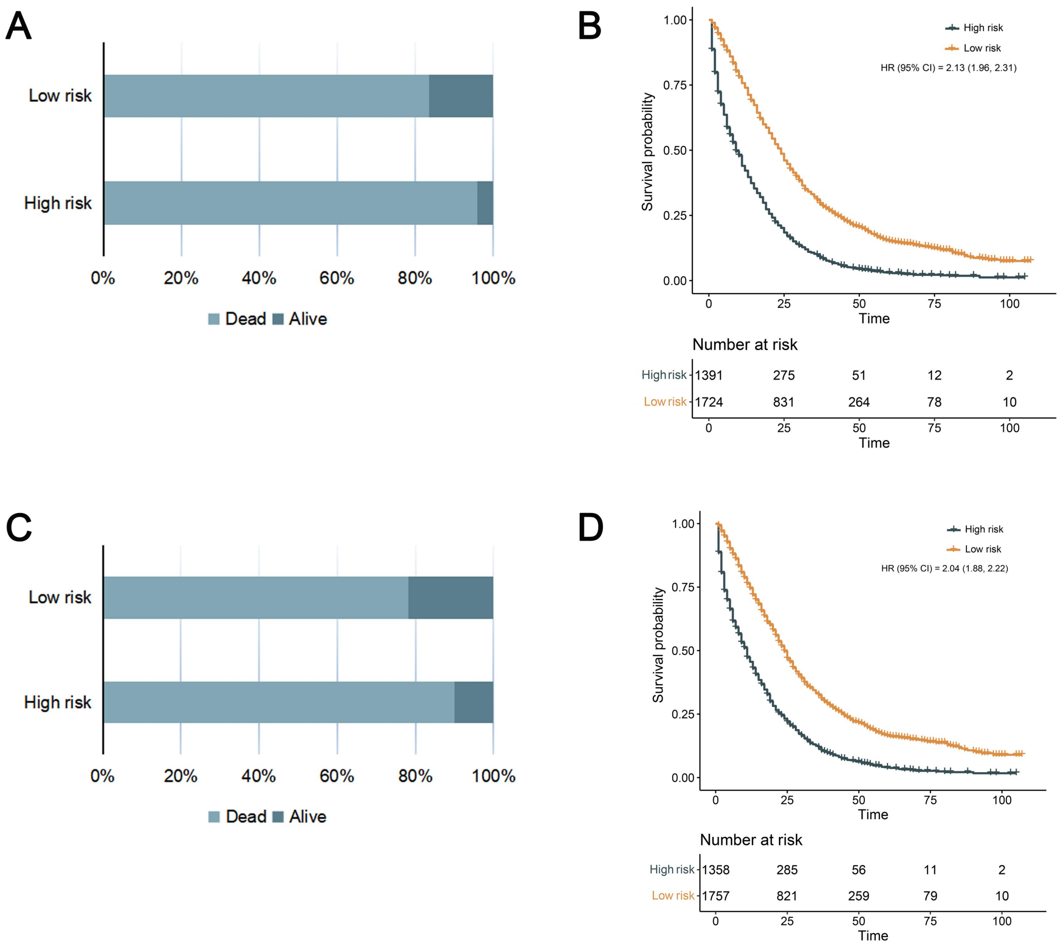


**Figure S4**. The OS (A) and CSS (C) status of 3115 CCLM patients between low-risk and high-risk groups in expanded testing set. Kaplan-Meier curves for OS (B) and CSS (D) of 3115 patients with CCLM between low-risk and high-risk groups in expanded testing set.
